# Integrating one-to-one peer support into psycho-oncological care in Germany: multi-perspective, mixed-methods evaluation of the isPO onco-guide service

**DOI:** 10.1007/s00432-023-04951-4

**Published:** 2023-06-05

**Authors:** Sandra Salm, Stefanie Houwaart, Natalia Cecon-Stabel, Antje Dresen, Holger Pfaff, Nadine Scholten, Theresia Krieger

**Affiliations:** 1 Faculty of Medicine, Faculty of Human Sciences, Institute of Medical Sociology, Health Services Research, and Rehabilitation Science (IMVR), University of Cologne, University Hospital Cologne, Eupener Str. 129, 50933 Cologne, Germany; 2 Institute of General Practice, Goethe University Frankfurt, Frankfurt, Germany; 3 House of the Cancer Patient Support Associations of Germany (HKSH-BV), Bonn, Germany; 4 Unit of Health Services Research in Childhood and Adolescence, Clinic of General Pediatrics, Neonatology and Pediatric Cardiology, University Hospital Düsseldorf, Medical Faculty, Heinrich-Heine University Düsseldorf, Düsseldorf, Germany; 5 Faculty of Medicine and University Hospital Cologne, Faculty of Human Sciences, Institute of Medical Sociology, Health Services Research, and Rehabilitation Science, Chair of Quality Development and Evaluation in Rehabilitation, University of Cologne, Cologne, Germany; 6 Center for Health Services Research, University of Cologne, Cologne, Germany; 7 Faculty of Medicine, Department of Medical Psychology, Neuropsychology and Gender Studies and Center for Neuropsychological Diagnostics and Intervention (CeNDI), University of Cologne, University Hospital Cologne, Cologne, Germany

**Keywords:** Peer support, Cancer, Psycho-oncology, Information provision, Evaluation, Mixed methods

## Abstract

**Purpose:**

One-to-one peer supporters called isPO onco-guides (isPO OGs) are an integral part of the new German psycho-oncological form of care ‘integrated, cross-sectoral Psycho-Oncology’ (isPO), additionally to professional care. The isPO OGs are cancer survivors with experiential knowledge, offering information on local support services and answering questions ‘all around cancer’ to newly diagnosed cancer patients. We aimed to evaluate the isPO OG service from three perspectives: patients, isPO OGs, and professional service providers.

**Methods:**

A mixed-methods approach was pursued. We conducted interviews and focus groups with the three person groups, and applied qualitative content analysis on the reported resources, processes and outcomes regarding the isPO OG service. Relations with patients’ utilisation and isPO OGs’ work satisfaction were identified with regression and correlation analyses of questionnaire and isPO care data. We compared isPO care networks (CN) with *X*^2^-tests or ANOVA. Qualitative and quantitative results were integrated during interpretation phase.

**Results:**

Qualitatively, the three person groups agreed on the benefits of the isPO OG service. The implementation’s maturity differed between the CN concerning established processes and resource availability. Attitudes of professional service providers appeared to be crucial for patients’ utilisation of the isPO OG service. Quantitative results emphasised the differences between the CN.

**Conclusion:**

Beyond differences in the CN, the isPO OG service has two psychosocial benefits: providing relevant, reliable, and understandable information; and offering the encouraging example that surviving and living with cancer is possible.

**Trial registration:**

The study was registered in the German Clinical Trials Register (No. DRKS00015326) on 30.10.2018.

**Supplementary Information:**

The online version contains supplementary material available at 10.1007/s00432-023-04951-4.

## Background

In addition to the physical burdens of medical treatment, cancer patients can be affected by psychological, social, and financial strains (Adler and Page 2008). It is estimated that 52% suffer from psychological distress (Mehnert et al. 2018). Moreover, cancer patients have high information needs throughout their trajectory (Goerling et al. 2020). Unfortunately, psychological and informational demands are the most unmet need domains in cancer patients (Fu et al. 2020; Henry et al. 2020; Karcioglu et al. 2023).

Since 2003, healthcare institutions in Germany have been able to gain a certificate as cancer centres from the German Cancer Society; accordingly, psycho-oncological care provision is required (Singer et al. 2013). Nevertheless, psycho-oncological services are still insufficiently integrated into routine cancer care. In 2008, the German Federal Ministry of Health set thirteen aims in the National Cancer Plan (NCP) (Bundesministerium für Gesundheit 2012). Aim no. 9 clarifies that ‘all cancer patients receive appropriate psycho-oncological care if needed’ (Bundesministerium für Gesundheit 2012). The NCP also demands that ‘for all cancer patients low-threshold, target-group specific, and quality assured information are available’ (aim no. 11b). Finally, in 2014, the German S3 guideline ‘Psycho-oncological diagnostics, counselling and treatment of adult cancer patients’ (Leitlinienprogramm Onkologie (Deutsche Krebsgesellschaft, Deutsche Krebshilfe, AWMF) 2014) was published. In addition to professional psycho-oncological care, the German NCP and guideline oblige that ‘peers are closely involved in care provision’ (NCP aim no. 7) (Bundesministerium für Gesundheit 2012), as peer support is considered an integral part of the psychosocial care of cancer patients (Leitlinienprogramm Onkologie (Deutsche Krebsgesellschaft, Deutsche Krebshilfe, AWMF) 2014).

### Peer support in cancer care

In healthcare, peer support is differentiated into different types like emotional, informational, and practical support (Dennis 2003; Gidugu et al. 2015; Simoni et al. 2011). Furthermore, peer interventions can be distinguished based on their aims, such as providing information as an education-based intervention (Simoni et al. 2011), facilitating health outcomes through social support (Gidugu et al. 2015; Simoni et al. 2011), or increasing self-efficacy so that patients are empowered to engage in beneficial health behaviours (Dennis 2003; Simoni et al. 2011). One of the most frequent peer support mechanisms is based on “experiential knowledge” (Proudfoot et al. 2012), which incorporates practical information and advice while authentically capturing the patient’s individual situation and needs. Peer supporters also differ from professional service providers in their ability to talk to patients in ways they understand (Proudfoot et al. 2012). Watson (2019) identified the ‘use of lived experience’ as the strongest mechanism of peer support. Sharing emotions makes patients feel understood, as they recognise that others have the same experiences, which facilitates hope (Gidugu et al. 2015; Watson 2019). Furthermore, shared experiences define peer supporters’ credibility and authenticity. Patients feel trust and a connection as long as communication with the peer supporter is on equal footing (Watson 2019), and, therefore, they can open up to the peer supporter (Gidugu et al. 2015).

Peer support for cancer patients is mostly done in face-to-face group settings (Ziegler et al. 2022). The offers found in the literature address especially breast cancer patients (Meyer et al. 2015; Ziegler et al. 2022). In Germany, a systematic integration of peer support into cancer care is still pending (Ziegler et al. 2022). Nevertheless, the psychosocial benefits of peer support for cancer patients are described similarly across different studies. It has been reported that patients gained experienced-based knowledge of cancer and its treatment (informational support) (Campbell et al. 2004; Dunn et al. 2003; Ussher et al. 2006; Ziegler et al. 2022), were more confident when interacting with professional service providers (Campbell et al. 2004; Ussher et al. 2006), felt less isolated (social support) (Campbell et al. 2004; Dunn et al. 2003), improved their coping strategies (Campbell et al. 2004; Dunn et al. 2003; Ussher et al. 2006; Ziegler et al. 2022), and experienced increased self-efficacy (Ussher et al. 2006; Ziegler et al. 2022).

Regarding the different types of peer support, Hoey et al. (2008) found one-to-one, face-to-face peer support to be one of the most effective peer support models. Systematic reviews concerning one-to-one peer support in cancer care and mental healthcare reveal high patient satisfaction (Macvean et al. 2008; Meyer et al. 2015; White et al. 2020). They also indicate that this type of intervention can reduce anxiety (Macvean et al. 2008) and emotional distress (Meyer et al. 2015) and increase empowerment (White et al. 2020).

Despite the requirements of the German NCP and the psycho-oncological guideline, and the evidence for the benefits of one-to-one peer support in cancer, the number of programmes that systematically include such support is quite limited in Germany. They are mostly restricted to offers in the inpatient sector (Slesina et al. 2014) or for adolescents and young adults (Richter et al. 2021; Stäudle and Lochbrunner 2019).

The new German form of care, ‘integrated, cross-sectoral Psycho-Oncology’ (isPO) (Kusch et al. 2022), uses one-to-one peer supporters called isPO onco-guides (isPO OGs) as an integral part of psycho-oncological care in order to fill this gap. In the isPO project (10/2017–03/2022), the eponymous new form of care was developed and implemented. It was comprehensively externally evaluated (prospective, formative, and summative) by a multidisciplinary team (Jenniches et al. 2020; Jenniches et al. 2020; Krieger et al. 2020, 2021a, b, 2022; Salm et al. 2022).

### The isPO onco-guide: a psycho-oncological one-to-one peer support

**Fig. 1:**
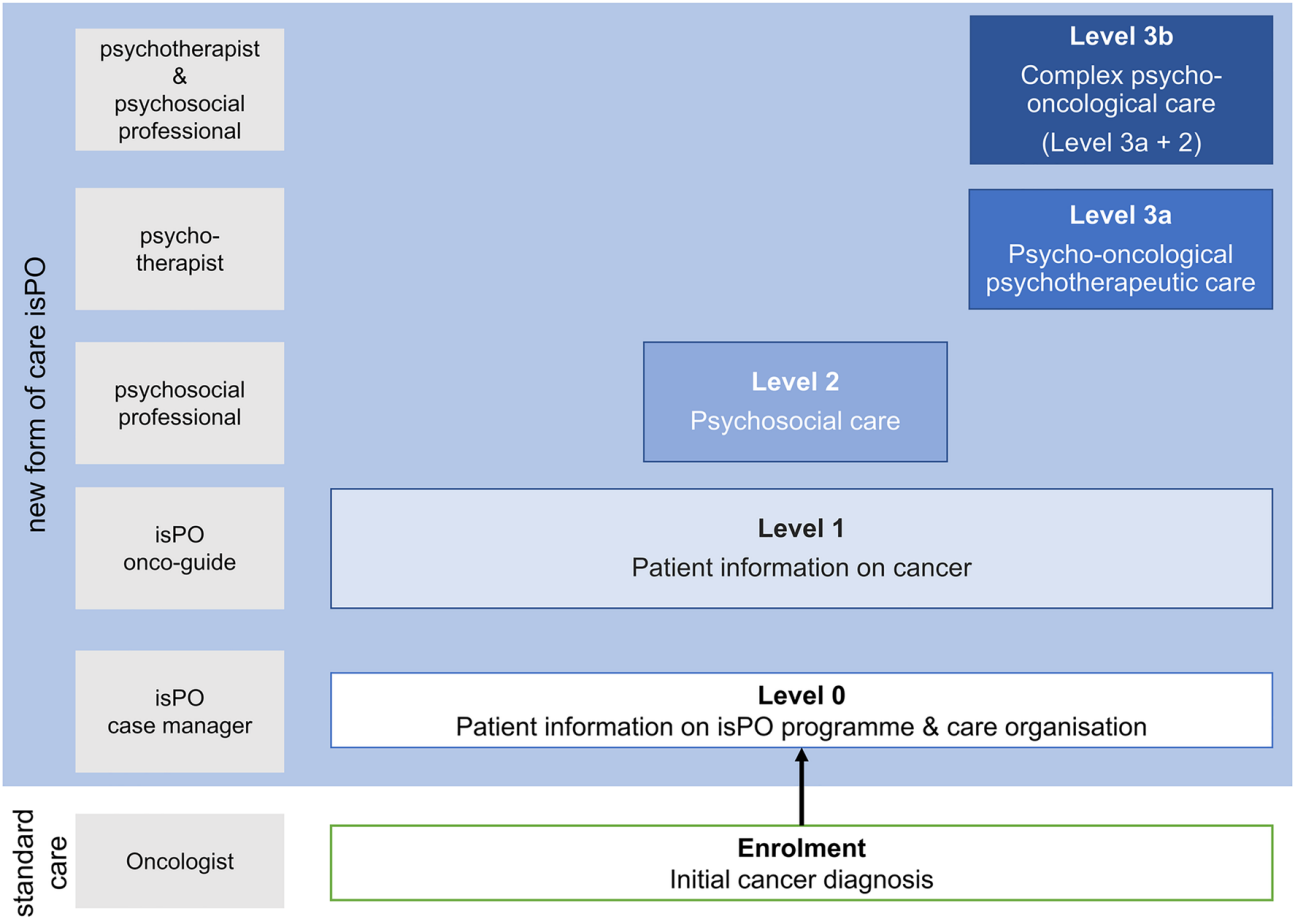
The isPO care levels and service providers. Adapted from Salm et al. 2022

Within isPO, newly diagnosed cancer patients receive psycho-oncological care for up to twelve months in a stepped-care approach. At the beginning of the care trajectory, every patient is assigned to a specific care level in accordance with the individual depression and anxiety level captured with the Hospital Anxiety and Depression Scale (HADS) (Zigmond and Snaith 1983) and the psychosocial care needs measured with the newly developed and validated psychosocial risks scale (Bussmann et al. 2022). Figure 1 displays the isPO care levels and their corresponding service provider groups.

**Fig. 2:**
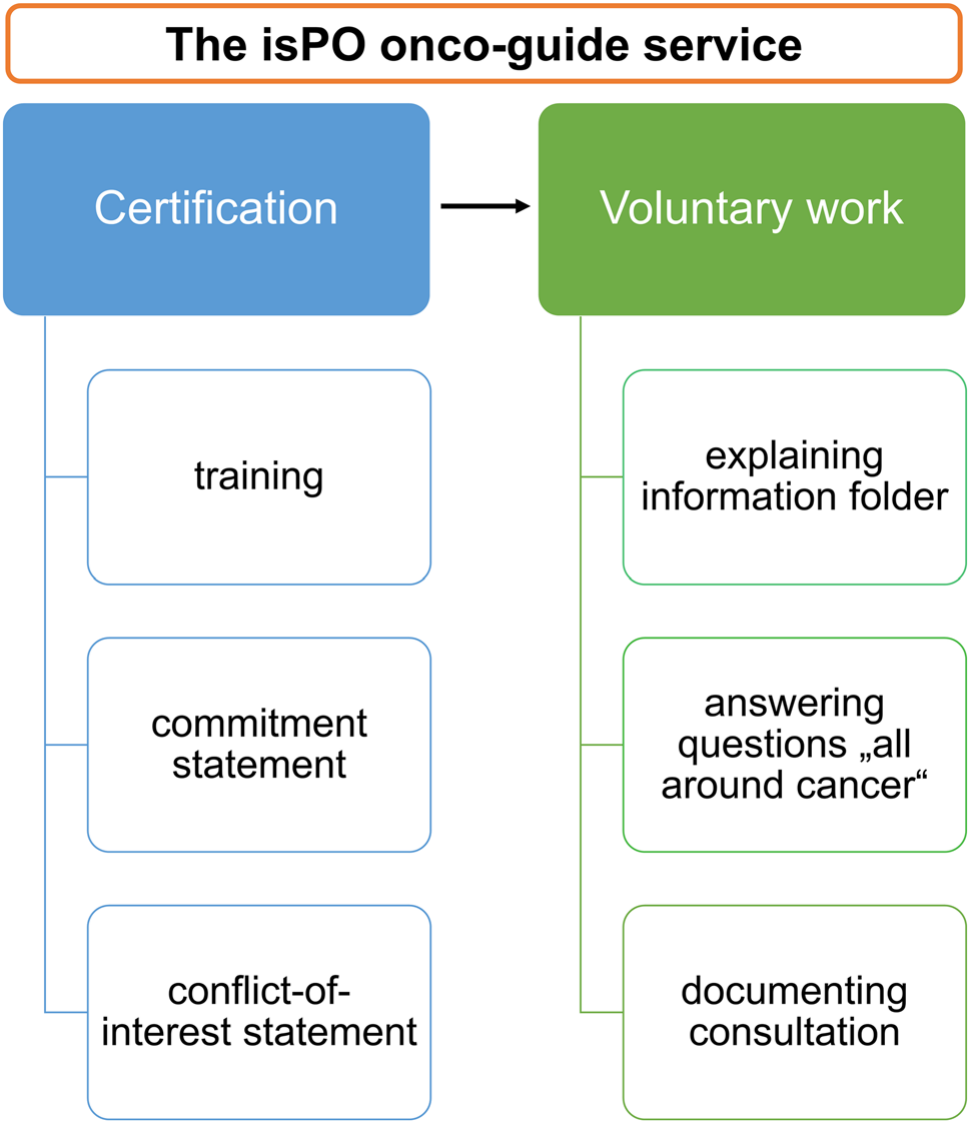
Overview of the isPO onco-guide certification requirements and voluntary work tasks

The new form of care isPO was implemented in January 2019 in four care networks in North Rhine-Westphalia, Germany. Every isPO care network comprises a cancer centre hospital and adjoint oncological outpatient practices. The hospitals differed in their location (metropolis, big city, middle city), their size (hospital of maximum care or standard care), and their teaching status (e.g. university hospital, academic teaching hospital). All of the hospitals offer acute care. Care level 1 is offered to every enrolled patient independent of the patient’s depression and anxiety level, which makes it a low-threshold service. It contains the provision of psychosocial support information by an isPO OG. isPO OGs are cancer survivors whose cancer treatment goes back at least one year. They are trained and certified for their voluntary work (Fig. 2). Services at levels 0, 2, 3a, and 3b are delivered by professional service providers (isPO case managers, psychosocial professionals, and psychotherapists) who are staff in the cancer centre hospitals of the care networks.

#### isPO OG certification

The isPO OG training consists of a five-hour seminar and covers four topics: (1) the new form of care isPO; (2) the role of the isPO OG; (3) exercises to facilitate conversation; and (4) documentation of the isPO OG consultations. The training was conducted by a patient representative of the cancer self-help umbrella organisation, House of Cancer Patient Support Associations of Germany (HKSH-BV), and a psychotherapist from the isPO development team. Aside from completing the training, cancer survivors must sign a commitment and a conflict-of-interest statement to obtain the certification as an isPO OG. The HKSH-BV oversees the acquisition, training, and certification, and was involved in developing the isPO OG care concept. The umbrella organisation forwards the data of certified isPO OGs to the respective isPO care network where the isPO OG wants to do their voluntary work.

#### isPO OG voluntary work

According to the isPO care concept, isPO OGs can offer up to two face-to-face consultations to every newly diagnosed cancer patient enrolled in the isPO programme. An isPO case manager who serves as a contact person for the patient throughout the 12 months in isPO coordinates the appointment between the patient and the isPO OG (Fig. 1). This consultation should be conducted in the cancer centre hospital as soon as possible after enrolment into isPO, and it should last a maximum of 45 min. The isPO case manager is also responsible for preparing the consultation-relevant documents. These consist of a conversation guideline and documentation sheet for the isPO OG and an information folder for the patient, which contains information on community-based psychosocial support services (according to the patient’s postal zip code), contact information for non-profit peer support groups, and services offered by the patient’s statutory health insurance.

During the consultation, the isPO OG provides the information folder and reviews it with the patient for a comprehensive orientation. Moreover, patients’ questions ‘all around cancer’ are answered except regarding medical and social legal advice. If the patient requests, the isPO OG may reflect upon their cancer care trajectory and experiences. In addition to providing information, the isPO OG acts as an authentic example that living with cancer is possible. Furthermore, they enable a conversation on equal footing. As the isPO OG service provides general information that may be relevant to all cancer patients, matching isPO OGs with patients with the same cancer type is not mandatory.

After the consultation, the isPO OG conducts the documentation on the prepared sheet and assesses their personal experience of the consultation’s quality. The written documentation is forwarded to the isPO case manager, who inputs the data into the isPO-specific IT documentation and assistance system CAPSYS^2020^. If no certified isPO OG is available for a specific appointment, an isPO case manager who has obtained the isPO OG training may conduct the appointment. This is also true for patients who do not want a consultation with an isPO OG, so information provision is secured for all patients.

### Objectives

Beyond the evaluations of one-to-one peer supports in cancer care in Germany (Richter et al. 2021; Slesina et al. 2014), we aimed to evaluate the isPO OG service as an integrated peer support within a psycho-oncological care programme. The evaluation design of the entire isPO programme and, thus, of the isPO OG service was based on the Medical Research Council framework for the evaluation of complex interventions (Jenniches et al. 2020; Moore et al. 2015; Salm et al. 2022). The focus is therefore on (1) implementation facilitators and barriers, feasibility and reach of the isPO OG service, (2) the acceptance and satisfaction of patients, isPO OGs, and professional service providers, (3) the different isPO care networks as the context, and (4) the health outcomes at patient level.

## Methods

**Fig. 3:**
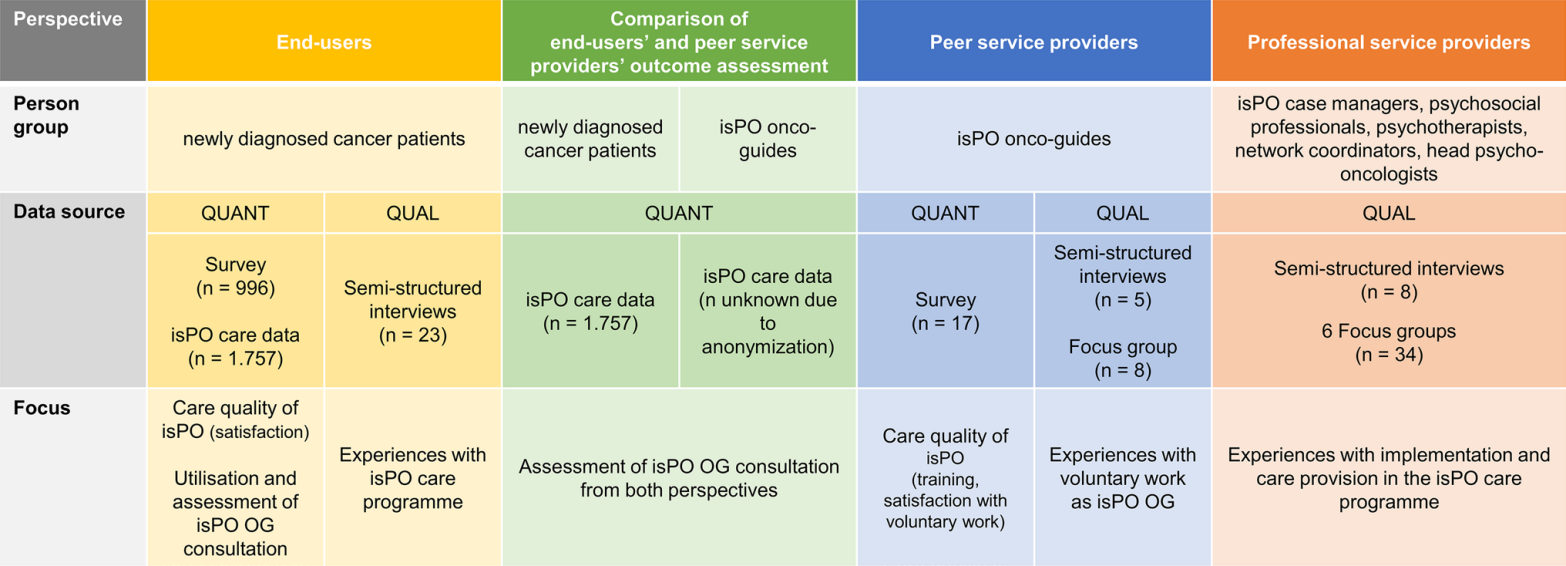
Data sources of the multi-perspective isPO onco-guide evaluation

The evaluation of the isPO OG service was part of the comprehensive evaluation of the overall isPO programme (Jenniches et al. 2020). Therefore, all the methods mentioned below—apart from data collections with the isPO OGs—were aimed at the isPO programme as a whole and not solely focused on the isPO OG service. The evaluation followed a mixed-methods approach with a concurrent qualitative and quantitative design (QUAL + QUANT) (Giddings and Grant 2006). The perspectives of three different groups were considered: (1) isPO patients (end-users), (2) isPO OGs (peer service providers), and (3) professional isPO service providers. Integrating multiple perspectives and methods permits thorough insights into the different experiences, opinions, and processes in implementation reality (Creswell and Plano Clark 2018; Curry and Nunez-Smith 2015; Greene et al. 1989). Different data sources on the isPO OG service were utilised for the three groups, as shown in Fig. 3.

Cancer patients could be enrolled into the isPO programme if they met the following inclusion criteria: new cancer diagnosis in one of the four isPO care networks, legal age ≥ 18 years, statutory health insurance, sufficient knowledge of the German language, and a clinical situation that enabled isPO care to be provided.

**Fig. 4:**
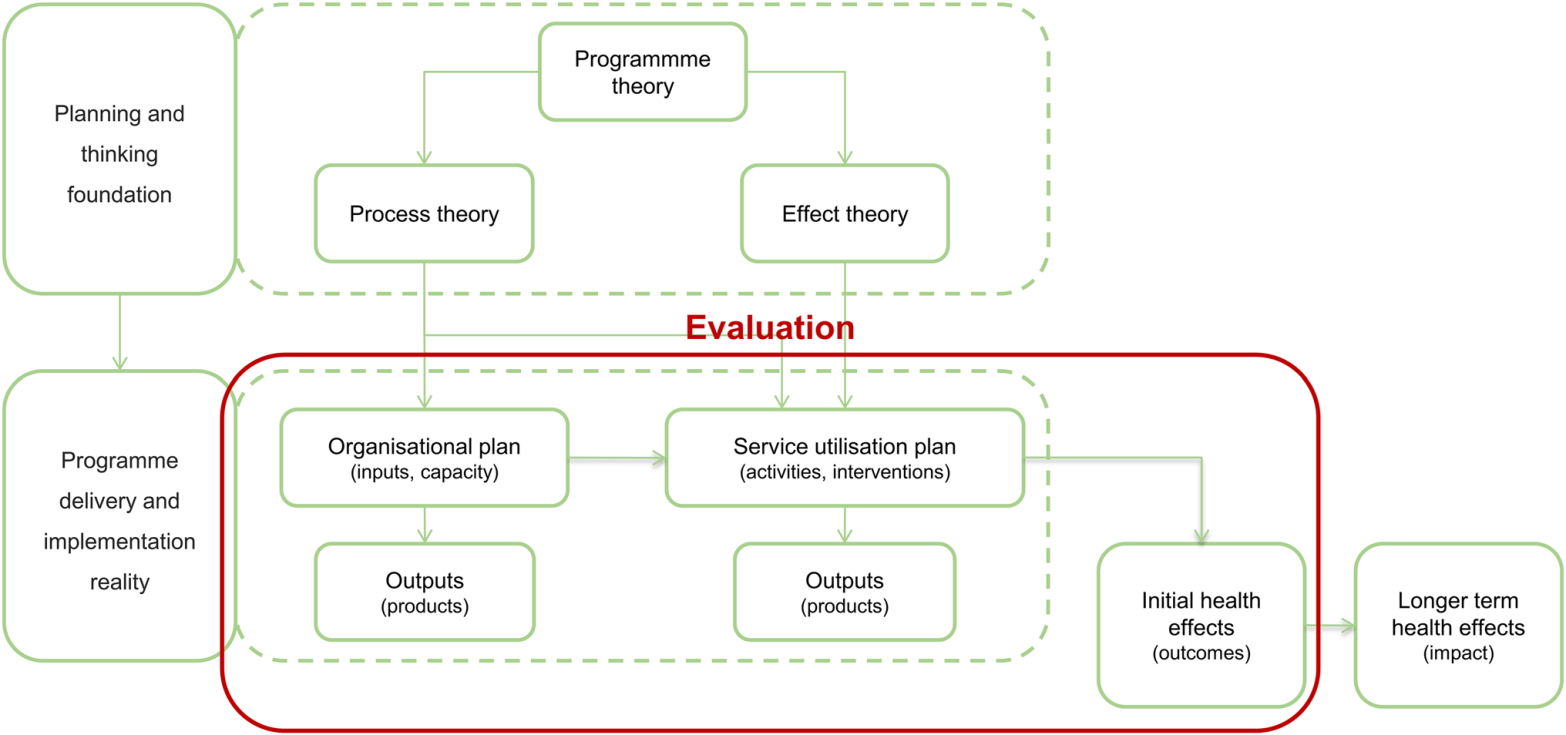
The programme theory of Issel and Wells with the embedded evaluation

The following will report the methodological procedures separately for the qualitative and quantitative methods. They were also analysed independently and will be compared and integrated into the discussion (Curry and Nunez-Smith 2015). The analysis and integration process were guided by the programme theory of Issel and Wells (2017) as the development of the new form of care isPO was based on this framework (Fig. 4).

According to Issel and Wells (2017), the evaluation of a health programme incorporates the assessment of (1) the resources that are required, for example, personnel (Organisational plan), (2) the processes needed to provide the care services like patient acquisition and work flows (Service utilisation plan), and (3) the health outcomes induced by care provision (Initial health effects). Moreover, the components ‘Organisational plan’ and ‘Service utilisation plan’ are divided into ‘Inputs’ and ‘Outputs’. Inputs are the resources and processes that are available, while outputs describe the results. An input at the level of ‘Organisational plan’ is, for example, the number of recruited staff; and a possible output is the work satisfaction. Regarding the ‘Service utilisation plan’, an exemplary input is the plan for care provision and an output might be the intervention coverage.

Results from qualitative and quantitative methods which belong to the same dimension were contrasted to examine if the results corroborate or contradict each other, or whether quantitative results can be explained with the help of qualitative findings.

### Qualitative methods

#### Sampling

The qualitative methods of the isPO OG evaluation contained the analysis of semi-structured interviews and focus groups with patients, isPO OGs, and professional isPO service providers. The acquisition of participants followed purposeful sampling (Patton 2002) according to the completion of the isPO care programme, the coverage of the isPO care networks, and the coverage of the different service provider roles. This means for the patient interviews that participants should have had already completed their 12-month isPO care trajectory. Moreover, participants should cover all four isPO care networks. This also applied to the interviews and the focus group with certified isPO OGs, the focus groups with professional isPO service providers on the care level (isPO case managers, psychosocial professionals, and psychotherapists), and the interviews with professional service providers on the managerial level (network coordinators and head psycho-oncologists of the isPO care networks).

Patients who were able to participate in an interview were acquired with the help of the care networks' psycho-oncologists. Then, the psycho-oncologists provided contact information for these selected patients to the isPO Trust Centre. The isPO Trust Centre ensured that the data exchange in the project was compliant with data protection regulations. It managed contact information of patients and service providers as well as pseudonyms for the different data sources to make data linkage possible. It was a unit within the institute responsible for the external evaluation of isPO but separated from the researchers both spatially and by personnel. For the interviews with isPO OGs, network coordinators and isPO case managers were asked to look for participants in their respective care network and provide their contact information. According to the isPO OG focus group, the HKSH-BV forwarded an invitation email from the evaluators to all certified isPO OGs so they could announce their participation to the isPO Trust Centre. The evaluators contacted the network coordinators and head psycho-oncologists directly. The network coordinators discussed within the team of the respective care network which members would participate in the focus groups with professional isPO service providers on care level and announced them to the evaluators. According to the purposeful sampling, it was requested that the participants cover professional service providers on all isPO care levels (isPO case managers, psychosocial professionals, and psychotherapists).

#### Data collection

**Fig. 5:**
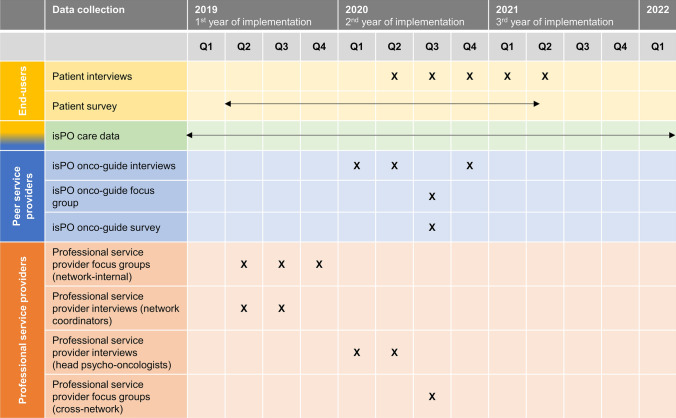
Time course of the data collections used for the isPO onco-guide evaluation

Data collection for the isPO OG evaluation started from the beginning of programme implementation. The time sequence of all data collection activities is illustrated in Fig. 5. All interviews and focus groups were conducted by the researchers of the external evaluation team. They were not involved in the development, implementation, and service provision concerning the isPO programme.

#### Patients

Patient interviews were conducted via telephone, as they took place during the COVID-19 pandemic in the second and third years of implementation. A semi-structured interview guideline was developed with the key topics ‘from diagnosis to participation in isPO’, ‘assessment of the care programme’, and ‘overall assessment’ (the key questions for all interview guidelines can be found in Online Resource 1). Four evaluators conducted the interviews, each responsible for one respective care network.

##### isPO onco-guides

Like the patient interviews, the interviews with isPO OGs were conducted via telephone by one evaluator (first author). A semi-structured interview guideline was used. The first author also functioned as head moderator of the isPO OG focus group and was supported by evaluation team members acting as co-moderator and recorder during data collection. The focus group took place in a seminar room in one of the care network hospitals. The same interview guideline was applied to the focus group and the telephone interviews. It contained questions on the topics of ‘admission and training’, ‘tasks and experiences’, ‘assessment of the isPO OG concept’, and ‘assessment of isPO’. The interviews and focus groups were conducted in the second year of implementation.

##### Professional service providers

For each of the four isPO care networks, a focus group with the network-internal professional service providers on the care level took place in the respective care network hospitals. Moreover, all four network coordinators were interviewed during an isPO quality workshop or in their network hospital. Two members of the evaluation team, acting as head and co-moderator, conducted these eight data collections within the first year of implementation. The semi-structured interview guidelines were similar and primarily contained questions on the implementation of the new form of care isPO and the communication and cooperation within isPO. The data collection described in the following occurred in the second year of implementation. The interviews with the four head psycho-oncologists occurred during the COVID-19 pandemic and were conducted via telephone. Topics of the interview guideline were ‘communication’, ‘assessment of the isPO care concept’, and ‘evaluation of changes due to the implementation of isPO’. The same evaluator conducted the four data collections per person group (professional service providers, network coordinators, and head psycho-oncologists).

Finally, one cross-network focus group each was performed with professional isPO service providers on the care level and the managerial level. Due to the COVID-19 pandemic and high workload, the participants could not travel on business for the focus groups. Therefore, they were conducted using video conferencing. In addition to the semi-structured interview guideline, a PowerPoint presentation was prepared with slides for each key question. The prepared topics were ‘experiences towards the implementability of isPO during the different phases’, ‘facilitators for implementation’, ‘barriers for implementation’, and ‘prerequisites for the dissemination of isPO into routine care’. Three evaluators conducted the focus group: (1) a head moderator, (2) a recorder who filled in the PowerPoint presentation with keywords from the participants’ statements, and (3) a technical supporter who also managed the chat posts.

Before every data collection, participants were informed about the procedure, and written consent was obtained. All interviews and focus groups described above were audiotaped. A professional service transcribed the audio material verbatim. For the focus groups, protocols for a change of speaker were created to facilitate the transcription. Before data analysis, transcripts were anonymised using replacements for names of persons and network locations; for example, *[name of psycho-oncologist]*.

#### Data analysis

Content analysis was performed for all transcripts of the interviews and focus groups (Krippendorff 2018) using MAXQDA. First, a deductive coding system was developed. The head codes were ‘Description of the isPO OG service’, ‘Facilitators’, ‘Barriers’, and ‘Suggestions for optimisation’. These four categories were, in turn, divided into the components of the programme theory of Issel and Wells (2017) for evaluation: ‘Organisational plan—Inputs’, ‘Organisational plan—Outputs’, ‘Service utilisation plan—Inputs’, ‘Service utilisation plan—Outputs’, and ‘Initial health outcomes’. Then, all text passages in the patient and service provider data material that refer to the isPO OG service were identified. One network-internal focus group with professional service providers, one interview with a network coordinator, and two interviews with head psycho-oncologists could not be considered for further analysis because they did not contain statements on the isPO OG service. Sub-codes were derived inductively from the identified text passages and classified into the programme theory-driven coding system. Concerning the isPO OG interviews and focus group, sub-codes of the already performed analyses for the formative evaluation of isPO were also used and classified in the aforementioned coding system. Two evaluators were involved in the data analysis, consenting the coding system each time after the data material was coded for a certain perspective and after the coding was conducted for all transcripts (see Online Resource 2). Coded text passages were condensed, and connections between Input and Output sub-codes were identified.

### Quantitative methods

#### Sampling

The quantitative methods considered the perspectives of isPO patients and isPO OGs.

All isPO patients who met the aforementioned inclusion criteria and gave consent to participate in the isPO study were considered for the evaluation. The network coordinators sent the contact information of all patients to the isPO Trust Centre on a monthly basis, as patients were enrolled into the isPO programme ongoing from 1/2019 to 3/2021.

Concerning the isPO OGs, all persons who obtained the training and had been certified were invited to the survey, regardless of whether they had already conducted conversations with patients (*n* = 45). As the HKSH-BV holds the contact information of all certified isPO OGs, it sent an invitation email to the isPO OGs on behalf of the evaluators. Persons who decided to participate in the survey shared their postal addresses with the isPO Trust Centre (*n* = 22).

#### Data collection

Three data sources were used for the quantitative methods of the isPO OG evaluation: 1) isPO care data of the IT documentation and assistance system CAPSYS^2020^, 2) survey data of patients (three months after enrolment), and 3) survey data of isPO OGs (18 months after implementation beginning). The survey data used were collected cross-sectionally, whereas the isPO care data are longitudinal and stem from the isPO care documentation (secondary data).

##### isPO care data

The isPO care data were extracted quarterly from the isPO IT documentation and assistance system CAPSYS^2020^ by the network coordinators and transferred encrypted to the isPO data warehouse at the Institute of Medical Statistics and Bioinformatics, University of Cologne. The data exports from the four care networks were merged at the isPO data warehouse and the data was comprehensively processed to make it usable for research purposes. The prepared isPO care data were again transmitted encrypted from the data warehouse to the evaluators. The isPO care data contain information on sociodemographic characteristics, psychosocial situation, psycho-oncological screenings (e.g. HADS (Zigmond and Snaith 1983)), and the isPO care trajectory of all isPO patients participating in the study who completed their 12-month care in isPO. The isPO care data also include evaluation data on the isPO OG service from the perspectives of isPO OGs and patients. The isPO OGs conduct their assessment right after an appointment together with the consultation documentation, which the isPO case manager inputs into CAPSYS^2020^. The patients receive a questionnaire by mail with the same items four months after enrolment during their intermediate psycho-oncological screening. After completion, they send the questionnaire back to their isPO case manager who enters the data into CAPSYS^2020^. The items concerning the isPO OG service have been newly developed and are guided by the three dimensions of sense of coherence (comprehensibility, manageability, and meaningfulness) (Antonovsky 1988). There is one item each for orientation, coping, and confidence.

##### Patient and isPO onco-guide survey

The surveys with patients and isPO OGs were conducted as paper–pencil questionnaires sent by the isPO Trust Centre to the participants along with a consent form. The questionnaire and consent form were filled in and returned to the Trust Centre in separate envelopes. According to Dillman’s Total Design Method (Dillman 1978), up to three contact attempts were made. The isPO patient survey three months after enrolment contained, among others, items on the care by the different service providers, including a newly developed scale concerning satisfaction with the isPO OG service. This scale showed an excellent internal consistency (Cronbach’s *α* = 0.904). Furthermore, two validated scales on social support were used: OSS-3 (Dalgard et al. 1995) and BS6 (Beutel et al. 2017). Due to pseudonymisation, isPO care and survey data could be linked as an exact matching (March et al. 2018).

The isPO OG survey was conducted 18 months after the implementation of the new form of care isPO into the care networks started. The questionnaire contained newly developed items on the training, the conditions of the voluntary work, and work satisfaction (according to the short scale on life satisfaction by Beierlein et al. (2014)), as well as a validated scale on the work-related sense of coherence (Work-SoC) (Vogt et al. 2013).

All survey questionnaires were scanned using the software Teleform to create SPSS data files. These pseudonymised data were transmitted to the evaluators for processing and analysis. The time sequence of the isPO care data collection and the surveys is shown in Fig. 5.

#### Data analysis

The following characteristic values describing the isPO OG service were computed from the variables of the isPO care data: percentage of patients who utilised the isPO OG service; date difference between enrolment and isPO OG consultation; percentage of patients who received an isPO OG consultation with a peer service provider; percentage of patients per reason isPO OG service was not conducted by a peer service provider; appropriateness of consultation timing; duration of the isPO OG consultation; percentage of patients who requested a second isPO OG consultation; to what extent the consultation helped in coping with cancer; to what extent it gave confidence; and to what extent it provided orientation.

For all these key figures, comparisons between the isPO care networks were conducted. X^2^-tests were computed for categorical variables and ANOVA for continuous variables. If variance homogeneity was not met, Welch statistics and Games-Howell post hoc tests were analysed.

Moreover, logistic regression analyses for the predication of utilisation were performed. Therefore, the isPO care data were linked with the patient survey data. The tested models were created according to the elements of Andersen’s model of health services use: predisposing characteristics, enabling resources, and needs (Andersen 1995).

Model 1 (predisposing factors) contains the variables of age group, gender, and educational level, according to the International Standard Classification of Education (United Nations Educational, Scientific and Cultural Organization 2012). We used age groups instead of an interval-scale age variable to identify possible non-linear relations.

Model 2 (enabling factors) consists of the predictor variables of partnership status, type of household, social support, and isPO care network.

Model 3 (need factors) takes into account tumour entity, depression and anxiety at the time of enrolment, and the assigned isPO care level.

For the items of the isPO OG assessment, which both patients and isPO OGs answered, paired t-tests were conducted to compare the perspectives. This covered the variables of the appropriateness of consultation timing, coping, confidence, and orientation.

From the patient survey, the scale ‘satisfaction with isPO OG consultation’ was analysed using ANOVA to compare isPO care networks.

The data of the isPO OG survey were analysed descriptively considering the volunteers’ activity status, their workload in hours per week, their subjective workload, satisfaction with training, and satisfaction with the voluntary work.

According to the latter, Spearman’s correlations were conducted for person-related and work-related variables. In the person-related analysis, age, gender, employment status, and level of previous experiences in cancer peer support were examined. The work-related analysis considered the objective and subjective workload, the number of consultations conducted, satisfaction with training, and work-related sense of coherence.

Analyses comparing the isPO care networks were not carried out for the isPO OG survey data, as the number of cases was insufficient.

To be able to present and interpret the results of the quantitative analyses according to Issel and Wells' programme theory (2017), the key figures described above were assigned to the programme theory components (Table 1).

**Table 1:** Key figures of the isPO onco-guide evaluation located into the programme theory components stratified after patients’ and isPO onco-guides’ perspective

	Component of programme theory				
	Organisational plan—Input	Organisational plan—Output	Service utilisation plan—Input	Service utilisation plan—Output	Initial health effects
Perspective			Key figures		
Patients	–	–	Utilisation rate Time span between enrolment and consultation Consultation with peer service provider Reasons for consultation without peer service provider	Timing of consultation Request for further consultation Satisfaction with consultation	Coping Confidence Orientation
isPO onco-guides	Activity status	Objective workload Subjective workload Satisfaction with training Satisfaction with voluntary work	–	Timing of consultation Duration of consultation	Coping Confidence Orientation

All analyses were conducted using IBM SPSS Statistics 28.

## Results

### Qualitative results

The results of the qualitative analysis are based on 23 interviews with patients, five interviews and one focus group (*n* = 8) with isPO OGs, and five interviews and five focus groups (*n* = 30) with professional isPO service providers. The interviews took approximately 30–90 min; the focus groups lasted 1.5–3 h. The ages of participating patients ranged from 32 to 65 years; 69.57% were female, and 30.43% were male. Participating isPO OGs were 42–69 years old; 69.23% were female, and 30.77% were male. Due to data protection requirements, sociodemographic characteristics of professional service providers were not collected.

### Organisational plan (Resources)

#### Acquisition, training, and certification

Most of the isPO OGs had previous experience in cancer self-help or patient representation. This was one of the motivations for becoming an isPO OG, but the aspect of giving something back was also important. One isPO OG recalled how difficult it was to find relevant information as a cancer patient; therefore, they would now like to help others. To be suitable for voluntary work as an isPO OG, one must be "*psychologically stable*" (isPO OG; Interview; ID1; care network 1) and have "*overcome one's cancer*" (isPO OG; Interview; ID3; care network 2). How the cancer survivors became aware of the isPO OG voluntary work opportunity are manifold and include mail distribution lists of self-help organisations, personal enquiry, or knowing an already active isPO OG.

Completing a training course is a basic requirement for the certification as an isPO OG. Many isPO OGs described the course as "*superficial*" (isPO OG; Focus group; ID; care network 1) and "*too short*" (isPO OG; Interview; ID7; care network 1), even though they stated that the provided conversation guideline was helpful. They desire the expansion of topics such as conducting a conversation and the provision of continuous training. The way patients and professional service providers reflect on the experience with the isPO OGs in their voluntary work makes their training satisfying.

#### Infrastructure and human resources

According to the isPO OGs, the infrastructure available differs depending on the care network. In care network 2, the isPO OGs have a designated room for consultations with prepared information folders in individual patient bags. In care networks 1 and 3, room facilities are scarce, which was also mentioned by the professional service providers, or the consultations take place in different hospital departments. In this case, the information folders are either deposited on the corresponding ward or must be collected from the isPO case management at a fixed location. Whether the isPO OGs receive an allowance for their work also depends on the care network. From care network 1, it was reported that no costs were covered; in care networks 2 and 3, travel costs were reimbursed, or a voucher for the staff canteen was handed out.

Patients and professional service providers of all care networks reported situations when no isPO OG was available for a consultation. Additionally, the isPO OGs asked for the expansion of the isPO OG teams in their respective care network so "*that one can provide even better support for every patient*" (isPO OG; Focus group; ID4; care network 3). From care network 4, it was reported that no isPO OG was active, although two persons were trained and certified. isPO case managers from care networks 3 and 4 described that they take over the isPO OG consultations on a regular basis.

#### Coordination and cooperation lines

According to professional service providers, isPO case management coordinates the scheduling of appointments between the patient and the isPO OG in most care networks (1, 2 and 3). As the isPO OGs are volunteers and not regular staff, WhatsApp groups were created out of necessity to facilitate this process. An isPO OG from care network 2 reported that they support the isPO case management as a contact person from the isPO OG team concerning the coordination. From this care network, the isPO OGs stated that they feel considered as part of the psycho-oncological team of the hospital:

“*So, we as isPO OG, we get along very well, but also [names of isPO case managers] - all is so well integrated. We don't feel like an annoying appendage that simply has to be done, but we really feel very valued. Also, when there are things to be done in the hospital, we are heard at least and can also give our voice to some extent”* (isPO OG; Interview; ID2; care network 2).

The professional service providers from care network 2 also reported that cooperation was established between the isPO case management and the isPO OGs. Furthermore, joint quality management circles have been conducted.

In care network 1, the team of isPO case managers is so large that the isPO OGs complained of not knowing every person and feeling less supported and valued. Therefore, they suggested that there should be a permanent contact person for the isPO OGs.

Another mentioned barrier for the cooperation between the isPO case management and isPO OGs in care network 3 was confusion about the distinct roles and tasks within isPO itself:

“*For a long time, we weren’t able to say concretely what we were going to do and when*” (Professional service provider; Focus group; ID1; care network 3).

This resulted in a sceptical attitude towards the isPO OG concept and its implementability in general, which also applies to care network 4.

At the level of the organisational plan, the isPO OGs of all care networks have repeatedly expressed the wish to increase the possibilities for exchange. This concerns, on the one hand, meetings with professional (psycho-)oncological service providers as well as (group) supervision with psycho-oncologists and, on the other hand, the exchange between the isPO OGs, which does not yet take place in an organised way.

Based on the results presented above, connections are made between the sub-codes of the categories 'Organisational plan—Input' and 'Organisational plan—Output'. These are summarised in Table 2.

**Table 2:** Identified consequences between ‘Organisational plan—Input’ and organisational plan—output’ sub-codes

Organisational plan—Input		Organisational plan—Output
Further training for isPO OGs	→	Accurate and continuous training
Number and availability of isPO OGs	→	isPO case manager acting as isPO OG
Cooperation lines between isPO OGs and professional service providers	→	Integration of isPO OGs in isPO service provider team
		Exchange between isPO OGs and isPO case managers
		Support of isPO OGs by professional service providers
		Optimisation of cooperation with professional service provider team
Lack of clarity on isPO OG role and tasks	→	Reservations of professional of service providers towards isPO OG service

#### Service utilisation plan (Processes)

#### Coordination

The isPO OGs describe different processes for the care networks regarding the coordination of consultations. In care network 1, the isPO case management calls the isPO OG to ask whether they can take over a particular appointment. In care network 2, an isPO OG as a representative receives the appointment requests from the isPO case management and forwards them to a WhatsApp group of the isPO OGs so that the volunteers can answer. In care network 3, the process is similar, but the isPO case management forwards the appointments to a WhatsApp group. The professional service providers from care network 2 report that their workflow is well established. According to the professional service providers from care network 3, the coordination of the isPO OG consultations is very time-consuming and "*costs effort*", so it is perceived as "*more efficient*" if the isPO case managers themselves carry out the "*consultations in 15 min*" with the patient (Service provider; Focus group; ID5; care network 3).

#### Timing, duration, and frequency

The timing of the isPO OG service within the care trajectory varies depending on the patient’s preference and understanding of this voluntary service offer; therefore, timing is perceived differently in its appropriateness. The isPO OGs report that most patients have already started biomedical oncological treatment before they receive the isPO OG consultation. From the patient's perspective, different timings were reported depending on the care network. In care networks 1 and 3, the consultation took place shortly before discharge from the hospital; in care network 2, at the beginning of oncological care. Particularly regarding information provision, the isPO OGs welcome the earliest possible timing as specified in the care concept. On the other hand, some patients report that the timing was too early for them to make contact with a peer and that they, therefore, rejected the isPO OG consultation. The professional service providers of care network 4 also mentioned that it was "*too much*" (Service provider; Interview; ID4; care network 4) for the patients.

Regarding the duration of the consultations, the isPO OGs of care networks 1, 2, and 3 state that they are at least 45 min and sometimes up to 90 min. According to the isPO OGs, adequate time is needed to understand the patient’s situation and needs. Therefore, the limitation to a maximum of 45 min, as stated in the training, is viewed critically.

The experiences of the isPO OGs are mixed as to whether, according to the concept, one or a maximum of two consultations are sufficient. It depends on the needs of the individual patient. Therefore, they suggest keeping the frequency of consultations flexible, similar to the duration.

#### Communication and understandability

Another reason patients refused to utilise the isPO OG consultation was that they did not understand what this care offer was about. For example, patients reported that they assumed it was a self-help group or that it was explicitly about the isPO OG sharing their experiences and medical history rather than providing general information. An important prerequisite is that the professional service providers inform the patients about the isPO OG consultation accordingly. Two patients from care network 4 stated that they were not informed about the isPO OG service at all. Additionally, it can be seen from the patients' descriptions that misunderstandings occur among the professional service providers, which are then passed on to the patients, leading to refusal:

"*It (Note: the isPO OG service) was introduced to me immediately at the first appointment by my counsellor, that this exists. She had, I think, two ladies, but they both had breast cancer and I have a completely different disease, so that it would probably not fit with me in terms of the topics we would talk about. She informed me about it and said that she could of course establish contact if I wanted to, but she also said that it probably wouldn't be a good fit in terms of the disease and the topics that we would deal with. I saw it the same way, so I didn't make use of it.”* (Patient; Interview; ID9_2; care network 4)

#### Utilisation experiences

When the isPO OG consultation takes place, patients appreciate that they are provided with information on support services as explained by the isPO OG. Several isPO OGs report that such a folder with relevant, reliable, and accurate information would have helped them at the time of their cancer diagnosis.

In this way, the isPO OGs act as authentic gatekeepers for psycho-oncological care and other support services for newly diagnosed cancer patients:

*"That this consultation is also the beginning for some people: ‘OK, to what extent can I perhaps open up or may I face my fears or what is coming up for me?’ That sometimes people don't even think about the possibilities beforehand and that through these consultations, this is consolidated or perhaps a bit of encouragement is given as to the direction in which it can go to get help.”* (isPO OG; Interview; ID4; care network 3)

For the isPO OGs, the consultation does not consist exclusively of the provision and explanation of the information folder, even though the isPO care level 1 is called "patient information". Rather, the information folder is the entry point into the consultation because it then *“often leads to very personal questions that are asked, or people tell us how they have been. So, it often becomes more personal, I think”* (isPO OG; Interview; ID4; care network 3).

By condensing the qualitative data to the level of 'Service utilisation plan' as just presented, the conclusions between ‘Input’ and ‘Output’ sub-codes shown in Table 3 are drawn.

**Table 3:** Identified consequences between ‘Service utilisation plan—input’ and ‘service utilisation plan—output’ sub-codes

Service utilisation plan—Input		Service utilisation plan—Output
Initiation and coordination of isPO consultation	→	Established workflow
		Service coordination
Predefined duration of consultation	→	Duration of consultation per patient
Frequency of consultations per patient (according to care concept)	→	Frequency of consultations per patient (as needed by patients)
Timing of isPO OG service/within care trajectory	→	Refusal by patients
Lack of understanding by professional service providers	→	Lack of understanding by patients
Missing information by professional service providers	→	Refusal by patients
Provision of relevant, reliable, and accurate information	→	isPO OGs as gatekeeper for psycho-oncological care
		Importance of information folder

#### Initial health effects

Through the isPO OG consultation, patients can talk to someone who has had authentic cancer experiences (peer). This brings the potential for new, supportive social contacts. Unlike professional service providers, the isPO OGs have their experiential knowledge, so the patients feel understood. As cancer survivors, the isPO OGs are an encouraging example that living and coping with cancer is possible. Patients, isPO OGs, and professional service providers shared this view (Table 4).

**Table 4:** Interview/focus group quotes from different perspectives regarding the category ‘Initial health effects’

Exemplifying quotes for the category ‘Initial health effects’		
Patients	isPO onco-guides	Professional service providers
“It was somehow very reassuring for me to see this person (*Note: the isPO OG*) in front of me, who you would never have thought had been through such a difficult time, who is just shining, looking healthy, being there with full concentration to conduct this consultation. That was just a very, very wonderful experience.” (ID2_2; care network 1)	“And I notice that they (*Note: the patients*) get a bit of hope, that they think ‘Ah, maybe I'll be lucky too. There's someone sitting here who has made it and why shouldn't I be able to achieve the same?’” (ID1; care network 1)	“’I recognise someone for whom it has moved on, so it can also move on well for me.’ That’s comforting to know.” (Focus group; ID3; care network 2)

### Quantitative results

In total, 1,757 newly diagnosed cancer patients participated in the isPO study. Their age ranged from 18 to 93 years (*M* = 57.34 years; *SD* = 13.36 years); 61.9% were female, and 38.1% were male. The majority of patients had an upper secondary education level (68.7%). The most frequently documented tumour entity was breast cancer (24.9%). Participant characteristics are presented in detail in Table 5. Of 1,599 patients invited, 994 took part in the survey (response rate: 62.2%).

**Table 5:** Characteristics of isPO patients per care network

Care network (CN)	Total (*n* = 1757)	CN 1 (*n* = 1036)	CN 2 (*n* = 235)	CN 3 (*n* = 257)	CN 4 (*n* = 229)
Characteristic	%	%	%	%	%
Age group (years)					
18–29	3.4	5.1	0.9	1.2	0.4
30–39	7.9	11.1	3.8	2.7	3.5
40–49	12.7	15.0	8.5	8.9	10.9
50–59	29.4	29.2	32.3	28.0	28.8
60–69	29.0	24.4	35.3	33.5	38.4
70–79	14.3	12.1	17.9	19.8	14.8
≥ 80	3.3	3.2	1.3	5.8	3.1
Gender					
Female	61.9	58.4	52.8	73.9	73.4
Male	38.1	41.6	47.2	26.1	26.6
Educational level					
Primary education	1.9	1.3	4.1	2.0	2.7
Lower secondary education	7.3	7.3	5.9	6.9	9.0
Upper secondary education	68.7	64.0	80.6	75.5	71.2
Bachelor or equivalent	6.7	7.6	2.3	6.1	8.1
Master or equivalent	14.0	18.2	6.3	8.6	8.1
Doctoral or equivalent	1.3	1.7	0.9	0.8	0.9
Tumour entity					
Head and neck	3.8	5.0	6.2	0.0	0.0
Oesophagus/stomach	6.4	9.4	1.8	3.2	0.0
Colon/rectum	6.0	4.9	7.5	9.7	5.4
Liver	1.2	1.4	0.9	2.0	0.0
Pancreas	2.7	3.7	2.6	0.8	0.0
Lung	10.1	5.2	22.9	25.4	2.0
Malignant melanoma	5.7	9.1	1.8	0.0	0.0
Breast	24.9	15.0	17.2	50.4	52.0
Female genital organs	5.7	6.2	2.2	4.4	8.4
Prostate	3.2	2.4	4.0	0.0	10.9
Kidney/urinary tract	1.1	0.5	1.8	0.0	5.0
Bladder	1.7	0.4	6.6	0.0	4.5
Haematologic malignancies	11.3	14.9	11.9	3.2	2.0
Other	16.2	21.9	12.8	0.8	9.9

Seventeen isPO OGs took part in the survey (response rate: 77.3%). The mean age was 56.35 years (SD = 10.65 years; range: 37–72 years). 70.6% of the participants identified as female, and 29.4% as male. Most isPO OGs were employed full time (41.2%) and have been actively involved in cancer self-help for 1–2 years (47.1%) (Table 6).

**Table 6:** Participant characteristics of the isPO onco-guide survey (*n* = 17)

Characteristic	*M* (SD) %
Age (years)	56.35 (10.65)
Gender	
Female	70.6
Male	29.4
Employment status	
Full-time employed	41.2
Part-time employed	5.9
Marginally employed (e.g. so-called 450 € job)	5.9
Retired due to reduced earning capacity	17.6
Retiree	29.4
Actively involved in cancer self-help for…	
< 1 year	11.8
1–2 years	47.1
3–5 years	11.8
6–10 years	17.6
> 10 years	11.8

#### Organisational plan (Resources)

Of the 17 certified isPO OGs who participated in the survey, 13 conducted consultations with isPO patients. This resulted in an activity status rate of 76.5%. The mean number of conducted consultations was 17.69 (*SD* = 14.14; range: 1–45). The isPO OGs report that they engage up to two hours per week in their voluntary work (92.9%; objective workload). Most of them state that they perceive this workload as “just right” (58.5%; subjective workload), while 5.9% assessed the workload as “too high” and 11.8% as “too low”.

A proportion of 76.5% would have liked more training for the isPO OG work. The item that asked whether the previous training was sufficient was answered as follows: 21.4% reported “little”, 64.3% “quite”, and 14.3% “totally”.

The scale of the satisfaction with the isPO OG voluntary work ranged from 1, “totally dissatisfied” to 10, “totally satisfied”. The mean value was 6.31 (*SD* = 2.81; range: 1–10).

Table 7 presents the results of the Spearman’s correlation analyses for work satisfaction with person-related and work-related variables.

**Table 7:** Results of Spearman’s correlation analyses of the isPO onco-guides’ work satisfaction and person-related and work-related variables

Variable	*r*_S_	*p*
Person-related variables		
Age	0.117	0.704
Gender	− 0.216	0.478
Employment status	0.142	0.642
Activity in cancer self-help	− 0.059	0.847
Work-related variables		
Objective workload (hours per week)	0.197	0.518
Subjective workload	0.354	0.235
Number of conducted consultations	0.011	0.971
Request for further training	0.175	0.568
Sufficiency of training	0.277	0.360
Work-related sense of coherence	0.618	0.024*
Work-related sense of coherence–comprehensibility	0.431	0.142
Work-related sense of coherence–meaningfulness	0.337	0.260
Work-related sense of coherence–manageability	0.625	0.022*

*Notes*. *p < 0.05

While no significant linear correlations could be observed for person-related variables, significant positive correlations were found with the overall work-related sense of coherence score and its manageability subscale. The higher the work-related sense of coherence and manageability, the higher the work satisfaction and vice versa.

#### Service utilisation plan (Processes)

The percentage of isPO patients who made use of the isPO OG consultation (utilisation rate) ranged from 61.9% (care network 1) to 96.6% (care network 2). Care network 2 was also the network with the highest rate of consultations conducted by an actual isPO OG peer service provider (84.1%); care network 4 had the lowest rate (3.4%). Across all care networks, the most frequent reason for a consultation not having been conducted by a peer service provider but instead by a professional service provider was “no isPO OG available” (86.0%). The highest rates were observed for care networks 3 and 4 (99.3% and 94.6%, respectively). It was documented that most patients did not request a further consultation (68.9%). The distribution across care networks was significant for all the aforementioned key figures with *p* < 0.001. Details on the analyses are presented in Table 8.

**Table 8:** Results of the *X*^2^-test and ANOVA for comparison of isPO care networks towards Service utilisation plan key figures

Care network (CN)	Total (*n* = 1757)	CN 1 (*n* = 1036)	CN 2 (*n* = 235)	CN 3 (*n* = 257)	CN 4 (*n* = 229)		
Key figure	%	%	%	%	%	Χ^2^	*p*
Utilisation rate	70.9	61.9	96.6	90.3	63.8	168.60	< 0.001
Consultation with peer service provider	*n* = 913	*n* = 343	*n* = 201	*n* = 224	*n* = 145	297.70	< 0.001
Yes	50.8	66.2	84.1	28.1	3.4		
No	49.2	33.8	15.9	71.9	96.6		
Reasons for consultation without peer service provider	*n* = 315	*n* = 35	*n* = 8	*n* = 142	*n* = 130	212.16	< 0.001
Peer service provider not wanted	14.0	91.4	50.0	0.7	5.4		
No isPO OG available	86.0	8.6	50.0	99.3	94.6		
Request for further consultation	*n* = 456	*n* = 219	*n* = 169	*n* = 63	*n* = 5	23.23	< 0.001
Yes	31.1	41.5	23.7	17.5	0.0		
No	68.9	58.4	76.3	82.5	100		
Key figure	M (SD)	M (SD)	M(SD)	M (SD)	M (SD)	Welch statistic	*p*
Time span between enrolment and consultation (days)	*n* = 1173 51.50 (58.54)	*n* = 591 69.12 (63.72)	*n* = 227 34.15 (45.31)	*n* = 219 51.62 (51.77)	*n* = 136 3.65 (8.51)	261.69	< 0.001
Duration of consultation (minutes)	*n* = 738 39.93 (19.86)	*n* = 308 44.00 (24.61)	*n* = 200 40.11 (16.11)	*n* = 98 40.79 (16.01)	*n* = 132 29.51 (8.10)	44.70	< 0.001
Satisfaction with consultation	*n* = 689 3.39 (0.60)	*n* = 356 3.38 (0.62)	*n* = 122 3.24 (0.65)	*n* = 143 3.49 (0.57)	*n* = 68 3.54 (0.44)	6.06	< 0.001

Comparisons of care networks were also significant for the continuous variables ‘Time span between enrolment and consultation’, ‘Duration of consultation’, and ‘Satisfaction with consultation (Table 8). Care network 4 had the lowest time span, with an average of 3.65 days, and care network 1 had the highest, with 69.12 days. Post hoc tests revealed significant differences between all care networks with *p* > 0.001. The average duration of consultation was lowest for care network 4 (29.51 min), which could also be observed in the post hoc tests in significant differences to the other three networks (*p* < 0.001 in each case). The newly developed scale on satisfaction with the consultation contains items with answers from 1 “totally disagree” to 4 “totally agree”. Mean values of the satisfaction scale ranged from 3.24 for care network 3 to 3.54 for care network 4. Post hoc tests revealed significant differences between care networks 1 and 4 (*p* = 0.040), care networks 2 and 3 (*p* = 0.007), and care networks 2 and 4 (*p* = 0.001).

In addition to the analyses described above, logistics regressions for the utilisation rate were computed with regression models according to Andersen’s model of health services use (Andersen 1995). Model 1 with predisposing factors was significant (*X*^2^ = 22.49; *p* = 0.004) with a variance explanation of Nagelkerke’s *R*^2^ = 0.019. Analyses of the odds ratios revealed gender and educational level as not significant, although die odds ratios of utilising the isPO OG consultation were at least two times higher for all age groups except > 80 years in comparison with the age group 18–29 years (Table 9).

**Table 9:** Results of the logistic regression analysis for the utilisation of the isPO onco-guide consultation

Regression models with predictors	OR	95% CI	*p*
Model 1: Predisposing factors (*n* = 1705)			
Age group (years)			
18–29^a^	–	–	–
30–39	2.03	1.08–3.82	0.028*
40–49	2.24	1.24–4.07	0.008*
50–59	2.77	1.59–4.83	< 0.001*
60–69	3.14	1.80–5.49	< 0.001*
70–79	2.33	1.29–4.20	0.005*
≥ 80	1.80	0.85–3.80	0.124
Gender			
Female^a^	–	–	–
Male	1.02	0.82–1.27	0.890
Educational level	0.97	0.91–1.03	0.334
Model 2: Enabling factors (*n* = 905)			
Partnership status			
Without a stable partnership^a^	–	–	–
In a stable partnership, living in the same household	0.95	0.46–1.95	0.889
In a stable partnership, not living in the same household	1.61	0.74–3.51	0.232
Type of household			
One-person household^a^	–	–	–
Couple/family household	1.14	0.55–2.37	0.729
Multi-family household/shared living	0.48	0.18–1.27	0.141
Other type of household	446,565,728.2	–	0.999
Social support			
OSS-3	1.03	0.95–1.11	0.530
BS6	0.98	0.94–1.03	0.504
Care network			
1^a^	–	–	–
2	821,995,742.5	–	0.995
3	16.24	6.53–40.38	< 0.001*
4	0.80	0.50–1.28	0.346
Model 3: Need factors (*n* = 1690)			
Tumour entity			
Head and neck	0.83	0.40–1.75	0.629
Oesophagus/stomach	0.34	0.18–0.62	< .001*
Colon/rectum^a^	–	–	–
Liver	0.43	0.16–1.19	0.104
Pancreas	0.63	0.29–1.39	0.253
Lung	1.02	0.55–1.86	0.962
Malignant melanoma	0.43	0.23–0.80	0.008*
Breast	0.99	0.58–1.69	0.968
Female genital organs	0.40	0.21–0.76	0.005*
Prostate	1.73	0.68–4.39	0.246
Kidney/urinary tract	0.76	0.25–2.37	0.638
Bladder	1.22	0.41–3.62	0.719
Haematologic malignancies	0.47	0.27–0.82	0.008*
Other	0.46	0.27–0.80	.005*
HADS total score	0.99	0.97–1.01	.349
isPO care level			
1^a^	–	–	–
2	0.99	0.63–1.54	0.960
3a	1.24	0.69–2.20	0.474
3b	0.91	0.50–1.63	0.740

Note. ^a^Reference category *p < 0.05

Model 2 with enabling factors was also significant and had the highest variance explanation of all three models (*X*^2^ = 164.84; *p* < 0.001; Nagelkerke’s *R*^2^ = 0.250). However, partnership status, type of household, and social support are not significant predictors. The odds ratio analysis showed a 16.24 times higher chance of utilisation for care network 3 than care network 1 (Table 9).

Model 3 (need factors) was significant as well (*X*^2^ = 72.78; *p* < 0.001; Nagelkerke’s *R*^2^ = 0.060). Although the HADS total score and the isPO care level were not significant predictors, the chances to utilise the isPO OG service were significantly lower for patients with the tumour entities oesophagus/stomach, malignant melanoma, female genital organs, haematologic malignancies, and others compared to the reference entity colon/rectum (Table 9).

The isPO OGs and the patients assessed the appropriateness of the timing of the consultation. The scale ranges from 0, “too early” to 2, “too late”. The mean value of the isPO OGs was slightly higher than those of the patients (*M* = 1.14 (*SD* = 0.42) and *M* = 1.05 (*SD* = 0.60), respectively). The paired *t*-test revealed the mean difference as significant (*t* = 2.62; *p* = 0.009).

#### Initial health effects

Along with questions regarding the timing of the consultation, patients and isPO OGs answered three assessment items concerning the consultation’s effects on coping, confidence, and orientation. The scales ranged from 0, “totally disagree” to 5, “totally agree”. For each of the three items, the isPO OG’s mean value was higher than the patient’s (Table 10). All paired t-tests were significant (*p* < 0.001).

**Table 10:** Results of the paired *t*-tests for isPO OG’s and patient’s assessment of the consultation effects

Variables	*M* (SD)	Mean difference	*t*	*p*
Coping–isPO OG	4.10 (0.91)	0.87	10.23	< 0.001
Coping–Patient	3.23 (1.40)			
Confidence–isPO OG	4.43 (0.83)	0.72	9.56	< 0.001
Confidence–Patient	3.71 (1.25)			
Orientation–isPO OG	4.02 (0.94)	0.56	6.60	< 0.001
Orientation–Patient	3.46 (1.43)			

## Discussion

This study aimed to comprehensively evaluate the isPO OG one-to-one peer support by capturing the perspectives of patients, isPO OGs, and professional service providers, following a mixed-methods approach and the programme theory model of Issel and Wells (2017). To our best knowledge, this is the first study that applied this approach and thus offered insightful knowledge regarding the benefit of one-to-one peer support for newly diagnosed patients.

**Fig. 6:**
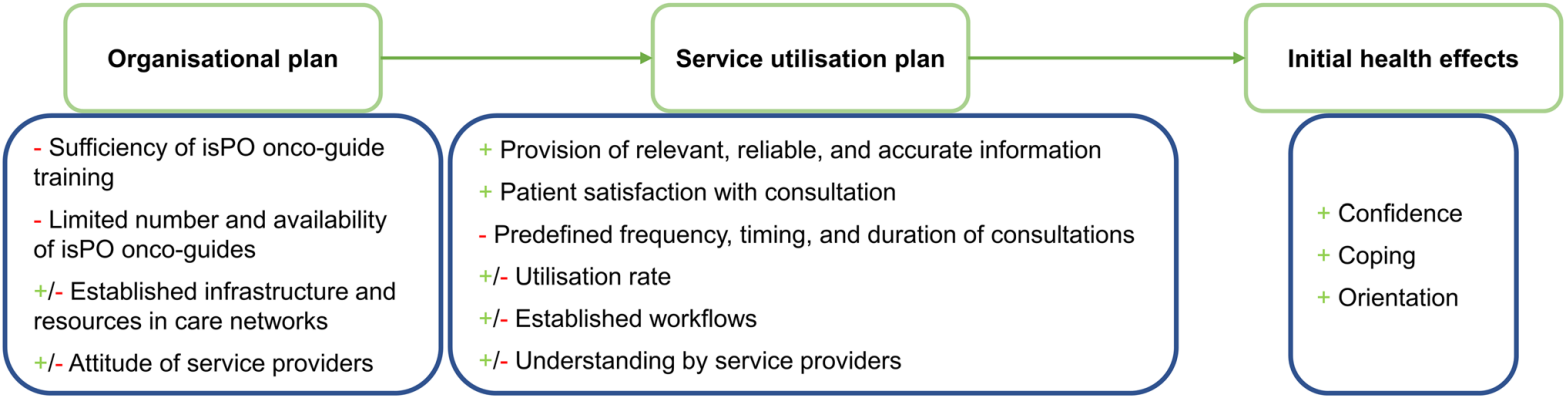
Condensed results of the isPO onco-guide evaluation

Figure 6 provides an overview of the positive and negative results of integrating the isPO OG service as one-to-one peer support according to the model components of Issel and Wells (2017).

### Organisational plan

The training for the isPO OGs was one of the relevant topics at the Organisational plan level. Both qualitative and quantitative results revealed that isPO OGs required more accurate and *comprehensive basic training*, particularly in facilitating conversation and deepening the topics in further courses (for example, differentiation from the concerns of the patients as a means of self-protection). The importance of the training was also underlined by the significant correlation between the work satisfaction of isPO OGs and the work-related sense of coherence scale manageability. Charles et al. (2021) identified in their systematic review the most relevant topics for initial training of peer supporters in mental healthcare, including, for example, communication and peer support of worker well-being. These results correspond to the demands of the isPO OGs and underline their importance. In addition, appropriate training helps to prevent volunteers from burnout so that they can pursue their work for longer and, thus, secure the service in the long term (Claxton-Oldfield 2016; Hopper and Skirton 2016). We recommend optimisation of the training curriculum due to the requirements in the field and ongoing supervision and intervention on a professional basis.

Across all perspectives and isPO care networks, there is agreement that the *size of the isPO OG teams* and the availability of volunteers for appointments are crucial factors for implementing the isPO OG service, which corresponds to one-to-one peer support programmes in the UK (Hughes et al. 2009; Pistrang et al. 2012). Thus, the participatory development of a suitable, context-specific acquisition strategy, as well as measures of outreach and commitment of the isPO OGs to the voluntary work, is of particular importance to secure isPO OG service provision to avoid high staff turnover in the isPO OG team and to distribute the workload among all volunteers (Treweek 2015). Regular and flexible supervision in the event of stressful patient conversations is considered helpful in this context (Sundram et al. 2018). Although the HKSH-BV, as a patient representative organisation, was assigned to the acquisition, among other things, its role, according to the project application, was to be exclusively advisory, which was also reflected in the approved personnel resources. We recommend allocating sufficient resources to these organisations to foster sustainability and make the service available to other care centres.

In contrast, the quantitative analyses revealed that some certified isPO OGs have not yet been appointed to any consultation. This may make them feel unappreciated, which could decrease their motivation to engage as an isPO OG in the future (Myrhøj et al. 2020). The ways of cooperation and the available *resources,* such as premises and allowances for the isPO OGs, are described very differently between the care networks. In care network 2, for example, the professional service providers have established a corresponding infrastructure. This may be due to their particular motivation and the convincing nature of the isPO OG service, as it was reported that the isPO OGs were involved in decisions towards implementation. Furthermore, joint quality management circles were conducted, which were not even prescribed by the isPO care concept. Such team building and decentralised decision-making strategies express the readiness for change in this care network (Vaishnavi et al. 2019). Nevertheless, it would have been necessary to involve the service providers in the development of the isPO programme to achieve a tailored implementation and sustainable changes in all four care networks (Skolarus and Sales 2015). We recommend establishing the isPO OG quality management circles introduced by care network 2 as a cross-network activity so that the isPO OGs and care networks can learn from each other to overcome challenges and reservations.

### Service utilisation plan

Based on the reports of the professional service providers and the patient descriptions of the information about the isPO OG service, it appears that in care network 4, the voluntary service was not understood correctly and not perceived as an integral part of the isPO care programme. The particularly low utilisation rate also indicates this. Identified misunderstandings referred, for example, to the fact that matching a patient and isPO OG with the same tumour entity is not intended since the aim is to pass on information relevant to all newly diagnosed cancer patients. Patients’ understanding of a complex intervention is considered a ‘prerequisite for decision-making’ (Buhse and Mühlhausen 2015). However, these misunderstandings about the isPO OG service may, in turn, lead to a sceptical attitude that influences whether and how patients are informed about the service. Consequently, care network 4 had a significantly lower utilisation rate than the other three isPO care networks. Furthermore, the period from enrolment to isPO OG consultation was significantly shorter in care network 4 because the isPO case managers took over consultations, and, therefore, no appointment coordination between the patient and isPO OG was required. However, this procedure is inadequate for a one-to-one peer support concept.

Since the attitude towards a complex intervention is a relevant implementation factor (Grimshaw et al. 2002), we recommend that the identification of respective barriers and facilitators and the involvement of the professional service providers should take place in the development phase. In this way, low commitment and resistance due to insufficient participation (Salm et al. 2022) and training (Fraser et al. 2009) might be mitigated, while a tailored and stepwise implementation will be fostered (Skolarus and Sales 2015).

Almost all other age groups had a significantly higher chance of utilising the isPO OG consultation than 18–29-year-olds. However, this age group is only represented to a very small extent in the sample. Another reason is that young adults may have specific questions and needs (Zebrack et al. 2014) that they would prefer to discuss with peers of the same age.

Patients with tumour entities with a significantly lower probability of using the isPO OG service were treated almost exclusively in care network 1. In this hospital, patients with particularly complex and demanding cases are treated. Moreover, it has a large coverage area and short lengths of stay, which may lead to patients being unable to use the isPO OG service depending on distance and mobility. An extension of the service to local outpatient options such as cancer counselling centres and online support via an app could be supportive.

The *timing* of the isPO OG consultation within their individual cancer care trajectory was an aspect that led to a refusal by some patients (Campbell et al. 2004; Kowitt et al. 2019). Patients explained that a conversation with a peer shortly after cancer diagnosis was “too early and therefore might not do any good”. They assumed that the isPO OG must necessarily share their medical history, although this only happens upon the patient's request. The isPO OGs were undecided about the “right” timing and pleaded for a flexible approach based on the needs of the patients, which Campbell et al. (2004) identified as a benefit of cancer peer support. We recommend that the *frequency and duration* of the conversations should also be handled in a flexible, patient-oriented manner.

Within the four care networks, we identified different implementation and normalisation processes caused by varying resources for the organisation and coordination of the isPO OG consultations. When a corresponding infrastructure was expanded, the coordination lines between the isPO OGs and the professional service providers were jointly experienced and adapted where necessary. These interactions and optimisation attempts led to a maturation of the isPO OG service as an integral part of the isPO care provision. If resources were low, the required processes were considered burdensome. In these care networks, the proportion of conversations conducted by an isPO case manager instead of a peer service provider is significantly higher. This demonstrates that implementation factors like ‘resource availability’ and ‘organisational change’ are interrelated (Vaishnavi et al. 2019). Therefore, we recommend establishing the necessary resources (personnel and infrastructure) to carry out the processes (Issel and Wells 2017).

### Initial health effects

At the level of *initial health effects*, there exists a high level of qualitative agreement among the perspectives and care networks; quantitatively, the isPO OGs rate the effects significantly higher than the patients. This could reflect the fact that isPO OGs evaluate the peer support service retrospectively, including their experiences from their care trajectory. At the same time, isPO patients are still close to their diagnosis, which is reminiscent of the response shift in patient-reported outcomes (Breetvelt and van Dam 1991; Hamidou et al. 2011). A different understanding of the isPO OG service is also important. In addition, the isPO OGs have several consultations as a basis for evaluation, which they may use for comparison. Overall, the decisive factor was that the isPO OGs are a living example that cancer can be overcome in such a way that even demanding volunteer work can be practised. In addition to authentic and plain information provision, encouragement is the central core of the isPO OG service. This was evidenced both qualitatively and quantitatively, as the assessment on the item "confidence" showed the highest mean value among patients and isPO OGs.

This puts the isPO OG service in line with the effects identified in systematic reviews of one-to-one peer support in cancer care and mental healthcare (Macvean et al. 2008; Meyer et al. 2015; White et al. 2020).

### Strengths and limitations

Applying a mixed-method study design resulted in several advantages. While the quantitative analyses provided a general view of experiences and opinions, the qualitative analyses helped to understand the underlying reasons and conditions. The different data sources supported the multiplicity of perspectives so that the isPO OGs and the patients and professionals service providers articulated their views and experiences. Furthermore, a multi-contextual understanding was gained, as all isPO care networks were considered, and their similarities and differences were elaborated. The ability to synthesise these results helped draw useful recommendations for actions.

The individual methods and data sources, respectively, contain strengths and limitations. Although the isPO care data contain representative data on the entire isPO care trajectory, they can also be afflicted with documentation gaps and errors. Moreover, the isPO OGs are obliged to conduct their assessment directly after the consultation, whereas the patients do so four months after enrolment in isPO. However, the data linkage with the patient survey data provided relevant information on the topics of satisfaction with the isPO OG consultation and social support. In addition, the quantitative patient data helped gain an overview of all patients, as they were not interviewed in focus groups, unlike the isPO OGs and professional service providers, and not all the patients interviewed had utilised an isPO OG consultation. The number of isPO OGs surveyed by questionnaire was very small in absolute terms. Hence, no parametric statistical evaluations were possible, but isPO was implemented in only four care networks during the project phase so that the population of isPO OGs is statistically small. No quantitative data on the isPO OG service was available for the professional service providers. Therefore, the qualitative data was a necessary complement to capture comprehensive insights from all perspectives. The qualitative data helped classify and explain the quantitative results. For example, the different utilisation rates in the care networks could be explained by the fact that, according to the qualitative investigations, there were different resources and attitudes on the part of the professional service providers, which in turn influenced the patient information on the isPO OG service and, thus, the utilisation decision of the patients.

The isPO programme was implemented in only four care networks with a limited number of service providers. This resulted in partly the same participants within the interviews and focus groups. However, they took part in qualitative data collections at different time points so changes between the different implementation phases could be reflected.

The mixed-methods design allowed for comprehensive evaluation (Borglin 2015). However, only one-group post-test data were available for the initial health effects. Thus, in the sense of Issel and Wells, this is an outcome documentation (Issel and Wells 2017). Since the isPO OG service is one of several care levels in the isPO programme, it is questionable to what extent an outcome evaluation only at the level of the isPO OGs would be meaningful due to interactions.

In addition, within the project duration of four years, isPO was not only developed but also implemented and optimised. This means that the isPO OG service only reached a high degree of maturity at the end of the project. Thus, short-term health effects could be observed, but not medium- and long-term ones. Moreover, depending on the assigned care level, patients took up further interventions in isPO, so it is very difficult to differentiate the effects of the individual interventions and, thus, also that of the isPO OG service after completion of the 12-month care. Therefore, qualitative data were also used to supplement the initial health effects key figures. For future projects on complex interventions, we recommend a two-step approach. First, a project should be carried out that includes implementation and formative evaluation. After reaching high maturity, the summative evaluation should take place as a separate project. For this, appropriate funding programmes are necessary that enable such an approach.

### Practical implications

The isPO OG consultation offers low-threshold psychosocial support for newly diagnosed cancer patients. The hope-giving and encouraging aspects empower patients and may have a positive impact on adherence to cancer treatment (Theofilou and Panagiotaki 2012). Thus, peer service providers may amplify the interconnection between patients and professional service providers (White et al. 2020). Besides the clinical benefits, the isPO OG service may be cost-effective. As the experiences of isPO OGs are the focus of their work, they need far less comprehensive training than professional service providers. Furthermore, they work as volunteers rather than paid staff. However, in order to retain the volunteers and, thus, ensure the sustainability of the isPO OG service as a whole, clear regulations on financial allowances for the isPO OGs are essential (Ormel et al. 2019).

Moreover, the isPO OG service complements the peer support of cancer self-help groups who are mostly entity-specific and can be attended throughout the cancer care trajectory. With the provided information, isPO OGs make patients aware of self-help group offers and, thus, encourage participation.

## Conclusions

The isPO OG service is an integral part of the new psycho-oncological form of care isPO. It is offered to newly diagnosed cancer patients on a voluntary basis and engages cancer survivors as peer service providers. The isPO OG consultations embody a low-threshold offer for information provision (orientation towards support services) and the gift of courage and hope to newly diagnosed patients. With these two main characteristics, the programme fulfils the requirements of the German NCP and psycho-oncological guideline (Bundesministerium für Gesundheit 2012; Leitlinienprogramm Onkologie (Deutsche Krebsgesellschaft, Deutsche Krebshilfe, AWMF) 2014). This gives the programme the potential to be implemented as an independent form of care.

Overall, the initial health effects were described as psychosocially beneficial. However, resource, process, and utilisation differences were detected within the isPO care networks. These indicate that both flexibility and adaptation of the care concept are required at the organisational level, provision, and individual level. The perfect conceptual fitting, timing, frequency, and duration of the consultations should follow the actual patient needs in everyday care.

To optimise new forms of care like the isPO OG service, we recommended early involvement and participation of all relevant stakeholders, including patients or their representatives, professional service providers at the care and managerial level, and the isPO OGs. With its positive outcomes and low-threshold healthcare offer, the isPO OG service can serve as a blueprint for one-to-one peer support in other healthcare domains, such as stroke. The key elements of such integrated peer support are providing relevant and accurate information and demonstrating how to overcome or live with a disease soon after the diagnosis.

## Supplementary Information

Below is the link to the electronic supplementary material.

Supplementary file1 (PDF 233 KB)

Supplementary file2 (PDF 94 KB)

## Data Availability

The datasets generated during and/or analysed during the current study are available from the corresponding author on reasonable request.
