# Supplementary material for: Integrating one-to-one peer support into psycho-oncological care in Germany: multi-perspective, mixed-methods evaluation of the isPO onco-guide service
Source: J Cancer Res Clin Oncol. 2023 Jun 5;149(12):10399–422. doi: 10.1007/s00432-023-04951-4 (PMC10240138; doi:10.1007/s00432-023-04951-4)
Supplement: Supplementary file 1 — Supplementary file1 (PDF 233 KB) [file 432_2023_4951_MOESM1_ESM.pdf]

Salm, Houwaart, Cecon-Stabel, Dresen, Pfaff, Scholten, Krieger. Integrating one-to-one peer support into psycho-oncological care in Germany: Multi-perspective, mixed-methods evaluation of the isPO onco-guide service. Journal of Cancer Research and Clinical Oncology

Coding system for interviews and focus groups with patients, isPO onco-guides (isPO OG), and professional isPO service providers

	Component of programme theory				
	Organisational Plan – Input	Organisational Plan – Output	Service Utilisation Plan – Input	Service Utilisation Plan – Output	Initial health effects
**Head codes**	**Sub-codes**				
**Description of the isPO OG service**	Previous experiences in peer support<br>Motivation<br>Requirements of potential isPO OG<br>Acquisition as isPO OG<br>Expectations towards the isPO OG work<br>Cooperation lines between isPO OG and professional service providers<br>Matching of patient and isPO OG	Amount of training<br>Topics/content of training<br>Time between certification and work as isPO OG<br>Reasons for not being occupied as isPO OG<br>Number of isPO OG<br>Infrastructure<br>Workload for isPO OG<br>Allowance for isPO OG work<br>Integration of isPO OG in professional isPO service provider team<br>Support of isPO OG by professional service providers<br>isPO case manager acting as isPO OG<br>Network-internal exchange between isPO OG<br>Focus of isPO OG work<br>Authentic empathy and experience level	Initiation and coordination of isPO OG consultation<br>Timing within care trajectory<br>Time between patient enrolment and isPO OG consultation<br>Preparation and follow-up of isPO OG consultation<br>Setting<br>Changes due to COVID-19 pandemic	Frequency of consultations per patient<br>Duration of consultation per patient<br>Content of isPO OG consultation<br>Importance of information folder for consultation<br>Subjective perception of patient needs<br>Consultations with presence of relatives<br>Attitude of patients towards isPO OG service<br>Developing openness as patient<br>Trust of patients through openness of isPO OG<br>Perceived benefits for isPO OG<br>Strain for isPO OG<br>Individual coping strategies for stressful consultations	Perceived benefits for patients<br>New social contact<br>Emotional empowerment
**Facilitators**	isPO-experienced patients as new isPO OG	Emotional stability of isPO OG<br>Training in conducting a conversation<br>Satisfaction with training<br>Established resources<br>Additional resources for psycho-oncological care<br>Allowance for isPO OG<br>Age matching<br>Atmosphere in isPO OG team<br>Exchange between isPO OG and isPO case managers	Frequency of consultations per patient<br>Timing within care trajectory<br>Composition of information folder<br>Provision of relevant, reliable, and accurate information<br>Flexibility in matching isPO OG and patients<br>Acceptance of patients<br>Acceptance of professional service providers	Established workflow<br>Optimisation of care quality<br>isPO OG as gatekeeper for psycho-oncological care<br>Presence of relatives	Feeling understood<br>Encouragement/empowerment
**Barriers**	Eligibility criteria for isPO OG<br>Lack of clarity on isPO OG role and tasks<br>Number and availability of isPO OG<br>Diversity of isPO OG for matching with patients<br>COVID-19 pandemic	Experience of isPO OG<br>Amount of training<br>Topics/content of training<br>Lack of resources<br>Resource expenditure for hospital<br>Working hours of staff and isPO OG<br>Compatibility with hospital structures<br>Allowance for isPO OG<br>Reservations of professional service providers towards isPO OG service<br>Support of isPO OG by professional service providers	Lack of understanding of professional service providers<br>Missing information by professional service providers<br>Competing with other peer support services<br>Timing of isPO OG service<br>Predefined duration of consultation<br>Availability of information material<br>Composition of information folder<br>Importance of information folder<br>Mobility of patients<br>Length of stay in hospital<br>COVID-19 pandemic	Refusal by patients<br>Lack of understanding of patients<br>Service coordination<br>Frequency of consultations per patient<br>Connection between patient and isPO OG<br>Focus on self-help group organization	Noticeability of effects
**Suggestions for optimisation**	Selection procedure for isPO OG<br>Further training for isPO OG<br>Expansion of isPO OG teams	Accurate and continuous training<br>Allowance for isPO OG<br>Increasing appreciation of isPO OG work<br>Regular (group) supervisions<br>Optimisation of cooperation with professional service provider team<br>Regular exchange between isPO OG	Content and design of information folder<br>Flexible consultation offers (timing, duration, frequency)<br>Establishing as consultation for patients	Support in transforming isPO OG information into action	-

Corresponding author: Sandra Salm, Institute of Medical Sociology, Health Services Research, and Rehabilitation Science, University of Cologne, Germany
sandra.salm@uk-koeln.de
