# Supplementary material for: Integrating one-to-one peer support into psycho-oncological care in Germany: multi-perspective, mixed-methods evaluation of the isPO onco-guide service
Source: J Cancer Res Clin Oncol. 2023 Jun 5;149(12):10399–422. doi: 10.1007/s00432-023-04951-4 (PMC10240138; doi:10.1007/s00432-023-04951-4)
Supplement: Supplementary file 2 — Supplementary file2 (PDF 94 KB) [file 432_2023_4951_MOESM2_ESM.pdf]

Key questions of the interviews and focus groups with patients, isPO onco-guides, and professional isPO service providers

**Interviews with patients who completed the isPO care programme**
Please briefly describe how you felt after you were diagnosed.
How did you first learn about the isPO programme?
How do you think patients should learn about the programme?
How would you describe isPO to a friend in short words?
In what ways have you received support within the isPO programme?
The isPO programme was newly implemented, i.e., the hospital had to establish new structures and procedures.<br>Did you ever feel that these changes were apparent in your contacts?
How professional did you perceive the various service providers in the isPO programme to be?
To what extent has isPO met your individual support needs?
To what extent did organisational challenges arise during your care process?
What did you particularly appreciate about the isPO programme?
How did you feel about the 12-month care period?
What did you find unusual, unpleasant or in need of improvement?
Looking back, would you say that participating in the isPO programme was beneficial for you?
What would you wish for isPO in the future?
Is psycho-oncological support needed beyond the one-year isPO programme?
**Interviews and focus group with isPO onco-guides**
To what extent have you made use of cancer peer support services yourself or been active in peer support outside of isPO?<br>How long have you been certified as an isPO onco-guide and how long have you been active in the field?<br>How many cancer patients have you supported (approximately) since then?<br>*Just for the interviews:* How do you evaluate the role of the isPO onco-guide or peer support as an integral part of psycho-oncological care?<br>*Just for the focus group:* What do you associate with isPO?
How did it come about that you became an isPO onco-guide in the first place? Please describe the path from learning about the isPO programme for the first time to your certification.
If you were to describe your work to a self-help colleague, how would you describe the schedule of a "typical working day" as an isPO onco-guide?
How do you experience the consultations with the patients?
Just for the focus group: How do you experience working an isPO onco-guide through Corona?
To what extent were your expectations of the work as an isPO onco-guide fulfilled?
Just for the interviews: Please comment on this statement: “Just passing on an information folder, anyone can do that. Why is the isPO onco-guide needed?”
How do you evaluate the innovative concept of the isPO onco-guide overall?
How do you evaluate isPO?
What wishes do you have for the training and design of the work as an isPO onco-guide?
What do you wish for the future of isPO?

Corresponding author: Sandra Salm, Institute of Medical Sociology, Health Services Research, and Rehabilitation Science, University of Cologne, Germany
sandra.salm@uk-koeln.de

**Interviews with network coordinators and network-internal focus groups with professional service providers**
What is your task and role in isPO?<br>*Just for network coordinators:* How do you evaluate the role of the network coordinator?<br>Please finish the sentence: "I expect of the isPO project…"
Just for professional service providers: From your point of view, how well was psycho-oncology organised in your institution before isPO?
For network coordinators: Please tell us about the preparations for the start of isPO care in your network.<br>*For professional service providers:* Please tell us how you experienced the preparations and the start of the isPO project.
How do you experience the implementability of the isPO programme so far?
For network coordinators: From your point of view, how is the cooperation with the project partners in isPO going?<br>*For professional service providers:* How do you experience the cooperation and communication in isPO?
For network coordinators: How do you assess the acceptance of the isPO programme among service providers and hospital management?<br>*For professional service providers:* How do you perceive the acceptance of the project in your network?
How would you evaluate the project overall?
How do you assess the potential of isPO to be transferred to routine care?
**Interviews with head psycho-oncologists**
What is your task and role in isPO?<br>How do you evaluate the role of the clinical head of isPO?
How do you experience the communication with the project staff involved in the development of the care concept and the care processes?
How do you experience the exchange at the quality workshops?
How would you explain isPO to a colleague?
What distinguishes isPO from other psycho-oncological care programmes?
How do you assess the maturity of the care concept for the respective care levels?
Care level 3 in the treatment manual is important for your work.<br>How would you rate the isPO treatment manual that was specifically developed for care level 3?
How do experience the utilisation of CAPSYS^{2020} in your daily work?
How do you assess the changes that have taken place through isPO?
If you think of the isPO programme as a whole, how would you end the following sentence?<br>I find isPO ...
In your opinion, what would still have to be changed in the care concept or the care processes in order to be able to implement the programme nationwide in Germany?
What would you wish for isPO from your profession's point of view?
**Cross-network focus groups with professional service providers**
What do you associate with isPO?
How would you rate isPO in the course of the project so far?
How do you experience the implementability of isPO in your care network in regard to facilitating factors?
How do you experience the implementability of isPO in your care network in regard to hindering factors?
How do you experience the implementability of isPO in your care network throughout Corona? (facilitating and hindering factors)
What would it take for isPO to be transferred to routine care?

Corresponding author: Sandra Salm, Institute of Medical Sociology, Health Services Research, and Rehabilitation Science, University of Cologne, Germany
sandra.salm@uk-koeln.de
